# Nonconventional 1,8-Diazafluoren-9-One Aggregates for Green Light Enhancement in Hybrid Biocompatible Media

**DOI:** 10.3390/ma15145012

**Published:** 2022-07-19

**Authors:** Aneta Lewkowicz, Mattia Pierpaoli, Katarzyna Walczewska-Szewc, Martyna Czarnomska, Piotr Bojarski, Robert Bogdanowicz, Stanisław Pogorzelski, Leszek Kułak, Jakub Karczewski

**Affiliations:** 1 Faculty of Mathematics, Physics, and Informatics, University of Gdansk, ul. Wita Stwosza 57, 80-308 Gdańsk, Poland; piotr.bojarski@ug.edu.pl; 2 Faculty of Electronics, Telecommunications, and Informatics, Gdańsk University of Technology, 11/12 Gabriela Narutowicza Street, 80-233 Gdańsk, Poland; mattia.pierpaoli@pg.edu.pl (M.P.); rbogdan@eti.pg.edu.pl (R.B.); stanislaw.pogorzelski@ug.edu.pl (S.P.); 3 Institute of Physics, Faculty of Physics, Astronomy and Informatics, Nicolaus Copernicus University in Toruń, ul. Grudziądzka 5, 87-100 Toruń, Poland; kszewc@fizyka.umk.pl; 4 Faculty of Law and Administration, University of Gdansk, ul. Jana Bażyńskiego 6, 80-309 Gdańsk, Poland; m.czarnomska591@studms.ug.edu.pl; 5 Faculty of Applied Physics, Gdańsk University of Technology, 11/12 Gabriela Narutowicza Street, 80-233 Gdańsk, Poland; leszek.kulak@pg.edu.pl; 6 Department of Technical Physics and Applied Mathematics, Gdańsk University of Technology, 11/12 Gabriela Narutowicza Street, 80-233 Gdańsk, Poland; jakkarcz@pg.edu.pl

**Keywords:** 1,8-diazafluoren-9-one, titanium dioxide, carbon nanowalls

## Abstract

Organic aggregates currently play a prominent role, mainly for their unique optoelectronic properties in the aggregated state. Such properties can be related to the aggregates’ structure and the molecular packing mode. In the literature, we have well-established models of H and J aggregates defined based on the molecular exciton model. However, unconventional aggregates, the most unrecognized forms, have been generating interest among researchers recently. Within unconventional aggregation, aggregation-induced emission systems (AIE) are considered. In the present work, we discuss the effect of the forming of unconventional aggregation together with the change in dye concentration on the surface energy characteristics of the materials. All materials were prepared as hybrid biocompatible thin films where the matrix is TiO_2_ or TiO_2_/carbon nanowalls (CNWs) with the incorporated dye in the form of 1,8-diazafluoren-9-one (DFO). Using the time-resolved emission spectra and the determination of surface parameters from contact angle measurements, we indicated the correlation between the changes in such parameters and the concentration of DFO dye in two types of TiO_2_ and TiO_2_/CNW structures. To examine the propensity of DFO for aggregation, the internal energy of the dye was assessed in several aggregate structures using Quantum chemistry calculations. The results emphasize that DFO is an attractive structure in the design of new fluorophores due to its low molecular weight, the presence of a nitrogen atom that provides good coordination properties, and the ability to form hydrogen bonds. Our studies show that when using suitable matrices, i.e., rigid media, it forms the preferred forms of aggregates in the excited state, characterized by high emission efficiency in the band maximum of around 550 nm.

## 1. Introduction

Titanium dioxide provides a suitable environment for many dyes due to its optimal spectroscopic properties, well described in the literature [1,2,3,4]. However, so far, there is no information on the surface properties of titanium dioxide in the form of thin films. Therefore, in this paper, we describe these properties in an unconventional way using the wetting angle and parameters calculated indirectly from the wetting angle. An additional advantage of this potential correlation model between the dye in the DFO matrix and the parameters calculated from the wetting angle is the introduction of one more variable into the thin films, namely carbon nanowalls. They play the role of the host for the dye molecules, but it is not yet known how their presence affects the surface energy parameters and surface interactions. The presented matrix provides a highly durable environment for dyes including DFO. In addition, we observe changes in the structure of the analyzed dye depending on the concentration of the dye and the type of matrix. It should be mentioned that various solid matrices, i.e., polymeric, hydrogels, monolayers, and molecular multilayers, as well as matrices obtained by the sol–gel process containing incorporated fluorophores, find numerous applications as drug delivery systems, active media for laser production, solar panel manufacturing, as parts of biosensors, and in many other areas [5,6,7]. Investigations of the influence of the environment are crucial given their wide range of applications in engineering, nanotechnology, medical, forensic, and pharmaceutical sciences, as well as in the food industry. One of the significant issues is the study of the type and properties of aggregates that fluorophores form in such matrices. Changes in the environment and other factors can weaken or strengthen the efficiency of the aggregation process, which is essential from the perspective of subsequent applications of these systems.

Numerous authors describe the process of aggregation of molecules in solutions and analyze the influence of various factors on aggregation processes [8,9,10,11,12,13,14,15,16,17,18]. Bojarski’s work showed, among other things, that an important factor affecting the probability of aggregate formation is the concentration of the dye, and from a practical point of view, one can speak of a limiting concentration below which aggregation of dye molecules is hardly visible and is considered insignificant. In the case of Rhodamine 6G in aqueous solutions, this concentration is as low as 10^−5^ [M], while for solutions in which glycerol is the solvent, the change in the absorption spectral profile is discernible at concentrations higher than 10^−2^ [M] [19].

According to the results presented in the above works, the size and structure of the aggregates strongly depend on the environment in which the molecule resides and the concentration of the dye. Other factors affect the aggregation process in different systems: the amounts of electrolytes, surfactants, additives, chelating agents, the chemical structure of the dye, and external factors such as temperature and pressure.

Nowadays, due to the diverse applications of materials containing pure dyes, quantitative and qualitative control of the aggregation process are important. The already known processes of aggregation in solution several decades ago have been translated recently into modern struggles with this process in advanced hybrid materials, which are rigid systems with specific internal structures. It is important to control the presence of H or J aggregates, related not only to the chemical structure of the molecule or its concentration but also to the specific surface area of the material under study. Tyurin and coworkers noted that when an organic dye is incorporated into an inorganic matrix such as titanium dioxide, the porosity and pore size of the matrix have a great influence on the aggregation process, which can disappear despite the high concentration of dye [20].

Currently, due to the diverse applications of pure dye materials, quantitative and qualitative control of the aggregation process are important. In addition, it is worth noting that aggregation processes are often overlooked in the interpretation of spectroscopic properties, as evidenced by current procedures for the visualization of dactyloscopic traces with DFO. DFO working solutions contain high concentrations of DFO, and in the process of visualization of dactyloscopic traces, not only DFO complexes with amino acids but also DFO aggregates are involved, as indicated by our previous work [21,22,23].

In the present work, time-resolved spectroscopy shows new opportunities to reveal short-lived (fast) processes that are not visible in stationary spectroscopy. The time evolution of the spectrum allows us to observe significant evolutions in the spectroscopic properties of the dye with the change in its concentration in the matrix and with the change in the matrix for the same dye. The results obtained with this ultrasensitive method lead us to another possibility, namely, an attempt to relate the changes in spectroscopic properties for short-lived processes together with the change in energy on the surface of matrices in the form of thin films.

Solid material wettability stands for a fundamental property revealing information on the surface architecture and chemical structure of the surface modified with adsorption, etching, and covering processes. In addition, wetting measurements are among the most commonly performed ones. They are simple and enable surface sensitive analysis with ca. 0.5–1 nm depths [24]. A set of the surface wettability parameters, i.e., the apparent surface free energy γ_SV_, 2D adhesive film pressure Π, and work of adhesion W_A_, were determined from Contact Angle Hysteresis (C_AH_) data to quantify the surface energetics of the solid substrata affected by surface treatment procedures using the approach developed in [25]. This formalism allows the surface wettability energetics parameters to be derived from only three measurable quantities: the surface tension of the probe liquid γ_LV_, and its advancing θ_A_ and receding θ_R_ contact angles leading to C_AH_, defined as C_AH_ = θ_A_ − θ_R_. So far, the C_AH_ methodology has been used to quantify the process of chemical and electrochemical surface modification of a boron-doped diamond electrode with organic molecules [26].

In this work, we will answer the following question: Do the concentration changes in the dye in a titanium dioxide matrix and a titanium dioxide–carbon nanowalls hybrid matrix correlate with the changes in the surface energy and the mode of interaction on the surface of these materials with the external environment? The answer is important for the application of these materials as luminescent or electrochemical probes, already proposed by us as selective to the presence of alpha-amino acids in the test environment, with applications in the detection of cancer markers or Friction Ridge Analysis.

Determining the surface interactions and those responsible for the surface reactivity of structures in thin films is fundamental to the proposal of thin films in the form of TiO_2_ and TiO_2_/CNW matrices as potential fluorescent and electrochemical probes.

## 2. Materials and Methods

The chemicals used in the work were as listed below:

- 1,8-diazafluoren-9-one (Sigma-Aldrich, Munich, Germany), and it was spectroscopically pure (dye content 99%).
- Titanium(IV) tetra(2-propanolate)-99.000% trace metals basis, poly(ethylene glycol) p-(1,1,3,3-tetramethylbutyl)-phenyl ether (Triton X-100), hydrochloric acid, propan-2-ol, and pentane-2,4-dione (Sigma-Aldrich, Munich, Germany).
- Ethanol (POCH Company, Gliwice, Poland). DFO/TiO_2_ thin films were obtained using the sol–gel method.

### 2.1. Growth of BCNW

First, 1 × 1 cm^2^ quartz substrates were cleaned by ultrasonication in acetone and DMF and subsequently pretreated by H_2_-rich plasma. Substrate pretreatment and nanowall growth were performed by a microwave plasma-assisted chemical vapor deposition (MWPECVD) system (SEKI Technotron AX5400S, Tokyo, Japan). Prior to the CVD process, quartz glasses were seeded by spin-coating in a water-based diamond slurry. The detailed procedures for BDD and BCNW can be found in our previous studies [27,28]. Briefly, BCNWs were grown at 700 °C with microwave power equal to 1300 W for 10 min, using a mixture of H_2_, CH_4_, B_2_H_6_, and N_2_.

### 2.2. DFO/TiO_2_ Sol–Gel Synthesis

The precursor solution DFO/TiO_2_ was obtained using titanium(IV) tetra(2-propanolate), propan-2-ol, Triton X-100, and hydrochloric acid (37%). Separately, DFO was dissolved in ethanol. Next, both solutions were mixed by vigorous stirring.

The prepared solution was applied to a microscope slide using the spin-coating technique (SCI-40 LOT, Oriel spin coater, Darmstadt, Germany). The obtained DFO concentrations in the sol of matrices were as follows: 2 × 10^−2^, 1 × 10^−2^, 2 × 10^−3^, 1 × 10^−3^, 1 × 10^−4^, and 1 × 10^−5^ [M].

The internal filter effect was eliminated by using optical densities of the thin films below 0.1. In addition, the thickness of the thin films was controlled by selecting the gelation time, which was optimized at 110 min.

### 2.3. Apparatus

The topography of the surface was studied using atomic force microscopy (AFM Nanosurf Easyscan 2, Nanosurf, Liestal, Switzerland) in the contact mode. The surface analysis of images was conducted using Gwyddion 2.47 software (Department of Nanometrology, Czech Metrology Institute, Brno, Czech Republic).

Spectroscopic ellipsometry (SE) studies were handled using a Jobin-Yvon UVISEL phase-modulated ellipsometer (HORIBA Jobin-Yvon Inc., Edison, Middlesex County, NJ, USA, over the 300–1100 nm wavelength range). DeltaPsi software (v. 2.4.3) (HORIBA, Kyoto, Japan) was used to estimate the dispersion of the refractive index n(λ) and thickness of the DFO/TiO_2_/CNW structures. They consist of CNW films deposited on fused silica covered by the DFO:TiO_2_ composite overlayer. Since CNW films show mixed *sp^3^/sp^2^* compositions, they were simulated by a standard amorphous model. The dispersion of the DFO:TiO_2_ nanocomposite was emulated by the Forouhi–Bloomer model, consistent with the Kramers–Kronig methodology, related to the amorphous and polycrystalline TiO_2_ behavior.

Time-resolved emission spectra were obtained using a pulsed spectrofluorometer (2501S Spectrograph, Bruker, Optics Inc., Billerica, MA, USA), as has been described previously [29].

As a probe liquid for C_AH_ (Contact Angle Hysteresis) measurements, deionized water (Millipore system; conductivity 0.05 μS cm^−1^) with a pH of 5.8 ± 0.1 and surface tension γ_LV_ = 71.2 ± 0.2 mN m^−1^ was used, and the CA measurements were carried out at room temperature T = 25 °C with ambient relative humidity of 43%. The contact angle hysteresis and drop shape analyses were performed with a tiltable plane [30]. The axisymmetric drop shape analysis profile (ADSA-P) technique was adopted to determine C_A_ from the sessile drops (4–6 mm in diameter) images. The ADSA-P laboratory-built set-up is described in detail elsewhere [31,32]. A CCD monochrome TAYAMA 1/3ʺ B/W CCD camera (Tayama, Tokyo, Japan) and an M501 magnifying USB microscope (Polypower, Taipei, Taiwan) horizontally oriented were chosen to take sessile drop side images. The images were analyzed with the ImageJ (National Institute of Health, USA) routine. After attaching the solid material sample (about 1.5 mm in thickness, cut into 20 × 20 squares) to the base plane, a liquid drop was deposited on the substratum using a variable volume pipette, and then the plane was slowly tilted (see Figure 1 in [9]). For each surface, 5–10 measurements were performed at different surface locations for the sample spatial homogeneity evaluation, and C_A_ data were averaged. When the drop began to move, the critical advancing and receding contact angles, θ_A_ and θ_R_, were measured from the side view images. Recent wettability studies of metal surfaces coated with paint layers reveal the significant role played by the sample surface roughness architecture and environment relative humidity [33].

### 2.4. Quantum Chemistry Calculations Details

Molecular systems of the monomer, dimers, and trimers in different configurations were built using Avogadro and VMD. Initial geometries of each system were chosen arbitrarily to cover the most probable cases (J aggregates, H aggregates, and three unconventional aggregates). Next, the geometry optimization of each system was carried out using the Orca quantum chemical software package [34], employing the B3LYP-D3 functional [35] with a DEF2-SVP basis set [34]. Using optimized geometries, the thermodynamic properties of each system were calculated, again using the B3LYP functional with RIJCOSX approximation and DEF2-TZVP basis set [34]. Vibrational analysis verified that each structure was a minimum, not the transition state or saddle point.

To assess the propensity of DFO to form aggregates with different geometries, we simply calculate the difference between the inner energy of the complex and the separate monomers:

*E = NE_monomer_ − E_N_*

Because of the high basis set used in calculations, we decided to neglect the BSSE correction.

## 3. Results

### 3.1. The Structural Profile of Thin Films

**Figure 2:**
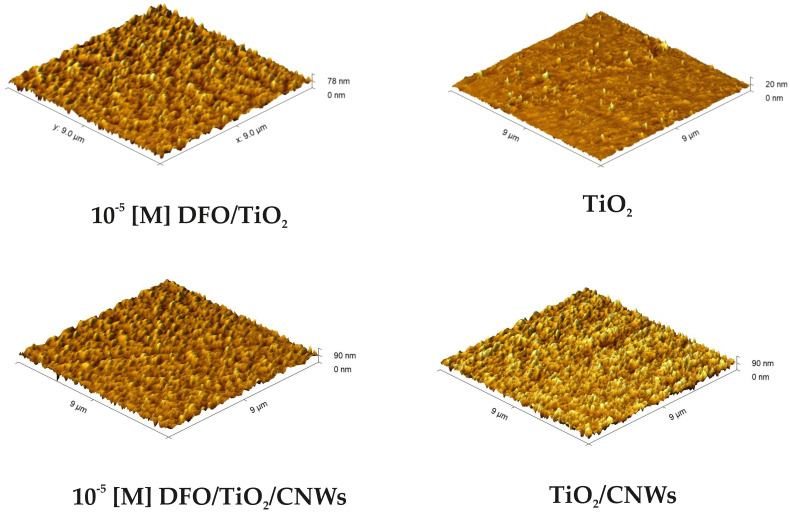
AFM image of thin films of DFO/TiO_2_ and DFO/TiO_2_/CNWs.

AFM topography images of DFO/TiO_2_ and DFO/TiO_2_/CNW thin films for the lowest DFO concentration analyzed are shown in Figure 2. The presented microscopic images are representative of the samples obtained. The structural analysis reported demonstrates that the resulting hybrid thin films are homogeneous with low roughness, as also suggested by our previous study [36]. In addition, it can be concluded that higher roughness is exhibited by TiO_2_/CNW thin films.

Ellipsometry is an established optical technique for measuring thin films and bulk materials. It uses changes in polarization due to reflection/transmission from a material’s structure to determine its properties, such as thickness and optical constant.

Table 1 summarizes the properties of fabricated DFO/TiO_2_/CNW structures. The DFO admixture in synergy with deposition on CNWs reveals high heterogeneity with strongly overestimated values of the refractive index. In addition, we found that the layer thickness increases with the addition of a spectroscopically active dye. The CNWs’ induction of the final surface morphology of the studied surfaces is obvious (see Figure 2).

An increase in the disorder of DFO/TiO_2_/CNW complexes and film thicknesses of the layers were evidenced by the large variation in the values of the obtained refractive indices. The derived ellipsometric results were compared with recently studied dyes in the TiO_2_ matrix [21]. In the case of TiO_2_, the surface structure is disordered, which will be reflected in further studies of surface properties.

### 3.2. The Spectroscopic Nature of Thin Films

**Figure 3:**
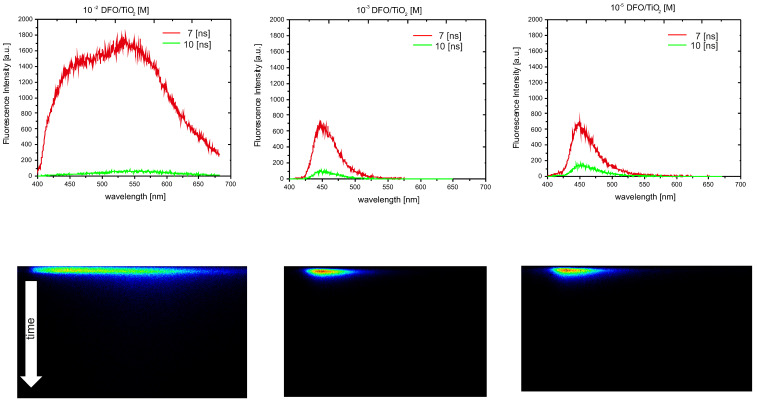
Time-resolved emission spectra of DFO/TiO_2_ thin films for different DFO concentrations: 10^−2^ [M] DFO/TiO_2_; 10^−3^ [M] DFO/TiO_2_; 10^−5^ [M] DFO/TiO_2_. The excitation wavelength was 380 nm.

**Figure 4:**
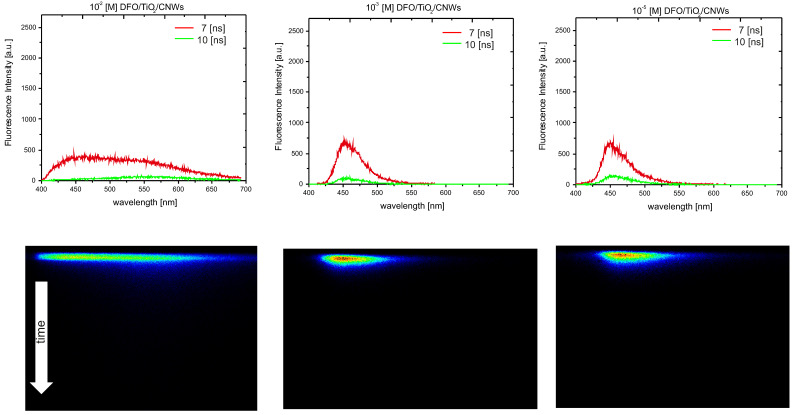
Time-resolved emission spectra of DFO/TiO_2_/CNW thin films for different DFO concentrations: 10^−2^ [M] DFO/TiO_2_; 10^−3^ [M] DFO/TiO_2_; 10^−5^ [M] DFO/TiO_2_. The excitation wavelength was 380 nm.

Using time-resolved fluorescence spectra analysis, a preliminary assessment of the spectroscopic properties of DFO structures in the TiO_2_ and TiO_2_/CNW matrices was performed. Figure 3 and Figure 4 show the time evolution of the fluorescence spectra for low and high concentrations of DFO in the TiO_2_ and TiO_2_/CNW matrix and original TRES images, respectively. The above figures demonstrate the changes in fluorescence intensity and the position of the band maximum with increasing DFO concentration. The limiting DFO concentration in the matrices for which the exclusive presence of DFO monomers can be assumed is 10^−3^ [M], while for 10^−2^ [M], significant broadening of the fluorescence band is observed, accompanied by the appearance of an additional band maximum on the long-wave side. This process is significantly stronger for DFO in titanium dioxide than for DFO in TiO_2_/CNW hybrid matrix.

The measurements are visualized as a quasi-triangular color plane image, with wavelength on the horizontal axis, time on the vertical axis, and intensity on the color scale. By cutting the image along the wavelength axis at a specific time after excitation, a fluorescence spectrum correlating with this time was obtained. The images were taken in short time windows, 10 [ns], which meant that we could observe short-lived processes invisible in stationary fluorescence measurements. In the 10 [ns] window, we observed significant changes for the highest concentration of DFO in the matrices, which also reflects the same dependence on the fluorescence spectra showing the time evolution of the fluorescence of the DFO dye in the various matrices.

Shortly after excitation, the fluorescence spectrum of the sample at the highest DFO concentration contained bands with maxima of 450 and 550 nm. This suggests that both monomer and aggregate emissions are observed. The evolution of the emission spectrum over time shows that the monomer emission at 460 nm gradually decreases, and only a broad band with a maximum at 550 nm is still present.

At the same time, these changes are much more pronounced for the TiO_2_ matrix.

**Figure 5:**
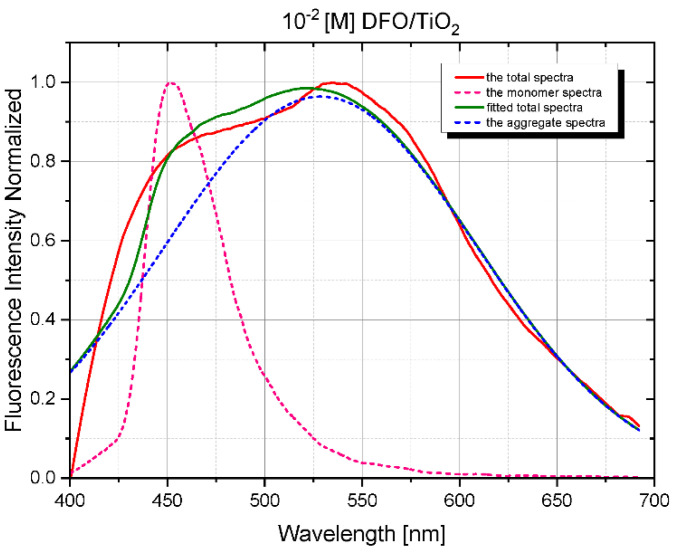
Numerically deconvoluted fluorescence spectra of DFO/TiO_2_ monomers and aggregates, the measured total fluorescence spectrum at *c* = 10^–2^ [M], and the numerical fit.

**Figure 6:**
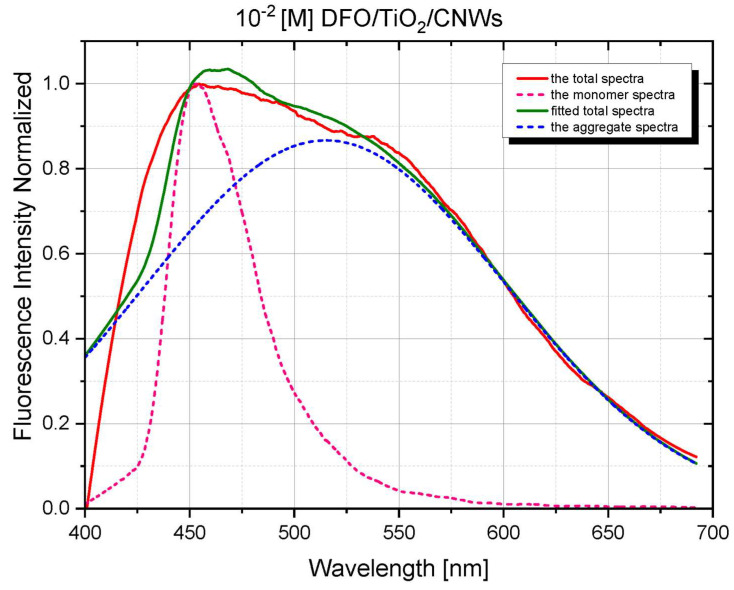
Numerically deconvoluted fluorescence spectra of DFO/TiO_2_/CNW monomers and aggregates, the measured total fluorescence spectrum at *c* = 10^–2^ [M], and the numerical fit.

Figure 5 and Figure 6 present numerically deconvoluted fluorescence spectra of DFO aggregates in TiO_2_ and TiO_2_/CNW films together with the monomer spectrum and an example of the fitted spectrum to the measured fluorescence spectrum at *c* =10^–2^ [M]. Calculations were based on our software using the procedures described previously [10,19]. It can be seen from the figure that the fluorescence spectrum of DFO aggregates is characterized by a significant increase in the half-width of the spectrum. This band probably contains a band of dimers and higher-order aggregates in the case studied.

**Figure 7:**
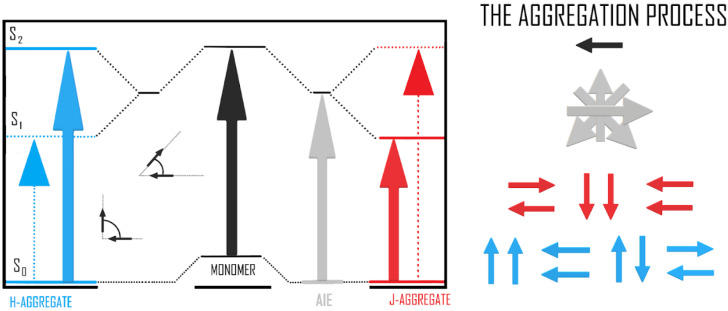
Energy scheme of excited states for various types of aggregates.

Changes in the structure of the DFO molecule are responsible for the observed concentration changes for the two matrices. In earlier work, we indicated the ability of DFO to aggregate [22,23]. In this work, we confirm this once again. At the same time, we move the interpretation towards the presence of unconventional aggregate structures that present the ability to fluoresce. Moreover, they enhance the emission of the considered structures. The literature gives examples of other molecules responsible for the formation of unconventional aggregates with the ability to emit fluorescence [37,38,39,40]. The energy diagram of such aggregates—aggregation-induced emission (AIE)—is represented in Figure 7.

AIE aggregates are structures that are π-conjugated organic molecules and show weak or negligible fluorescence in dilute solution, but in solid, rigid matrices, we observe enhanced fluorescence from these structures [41,42,43]. DFO is among the molecules characterized by the π-conjugated organic molecules. It is worth mentioning that a variety of intermolecular interactions (i.e., C⋅⋅⋅⋅O, O⋅⋅⋅⋅O, C-H⋅⋅⋅⋅O, C-H⋅⋅⋅⋅C) are the major interactions that provide a natural drive to fix the corresponding conformations of molecular aggregates and lead to the chromophore in the solid state being highly emissive. In the case of DFO in TiO_2_ and TiO_2_/CNW matrices, we observe the formation of highly emissive dyes (from 450 nm to 550 nm). In turn, computer calculations allowed us to identify the most stable aggregate conformations and directly relate these results to the interpretation of parameters characterizing the surface energies of DFO thin films in the two matrix types: TiO_2_ and TiO_2_/CNWs.

### 3.3. The Surface Energy Properties—Liquid/Solid Wettability Energetics

The most solid surface free energy determination formalisms rely on Young’s equation employing equilibrium C_A_ data [44]. In contrast, the C_AH_ model developed by Chibowski [25] allows the solid surface free energy γ_SV_ and the related surface wettability parameters of liquid–solid surface interaction energetics to be determined from three measurable quantities: the surface tension of probe liquid γ_LV_, and the dynamic contact angles θ_A_ and θ_R_. Particularly, the surface energy of a solid, γ_SV_, is calculated as follows [25]:

γ_SV_ = Π (1 + cos θ_A_)2/[(1 + cos θ_R_)2 − (1 + cos θ_A_)2] (1)

where the interfacial adsorbed liquid film 2D pressure, Π, is defined as:

Π = γ_LV_ (cos θ_R_ − cos θ_A_) (2)

Two-dimensional adsorptive film pressure П, according to the Gibbs adsorption theory [45], is related to the surface adsorption Γ (Gibbs excess) ~ П/RT, where R is the gas constant and T is the absolute temperature. For the one-molecule thick adsorption layer Langmuir model, the limiting area A_lim_ is occupied at the surface by the adsorbate unit: A_lim_ = 1/ΓN_A_, where N_A_ is the Avogadro number.

The surface-mediated C_AH_ should be related to the work of spreading W_S_ of liquid on a solid surface, which can be calculated from the work of adhesion W_A_ and the work of cohesion W_C_:

W_S_ = W_A_ − W_C_ (3)

where W_A_ = γ_LV_ (1 + cos θ_A_), and W_C_ = 2 γ_LV_ [45]. W_S_ is a thermodynamic quantity that relates the wettability to the mechanical strength of adhesion. It allows characterizing the competition between liquid/solid adhesions with a variety of liquids or substrata differing in their polarities [46].

The C_AH_ phenomenon can result from several effects: surface roughness, microscopic chemical heterogeneity, surface adsorption and molecular reorientation of the adsorbate material, and the liquid molecules’ penetration into the solid surface. It should be noted that for “non-hysteresis” systems (unlikely to be found in nature), where C_AH_ = 0 and θ_A_ = θ_R_ = θ_Y_, Equation (1) reads as [47]:

γ_SV_ = γ_LV_ (1 + cos θ_A_)/2 = W_A_/2 (4)

The surface free energy dispersive component γ_SVd_ is given by:

γ_SVd_ = γ_LV_ (1 + cos θ_A_)2/4 (5)

Dispersion (known as London) interactions between molecules of water and apolar components appear to be relatively strong and essential. These forces are responsible for the orientation of molecules adsorbed on the surfaces [45]. From the point of view of surface energetics, in the polymer (PMMA)–water solution interactions, forces of dispersive nature prevail, where the dissipative term γ_SV_d (Equation (5)) in the total free energy γ_SV_ accounts for up to 0.88–0.97% [30]. In the case of more complex water mixtures, particular values of the wettability parameters can be dependent on pH and ionic strength of the water phase since other intermolecular forces, such as hydrogen bonding, Lewis acid–base, electrostatic, etc., can play a significant role.

Surface energy is fundamental in controlling surface properties, so we show effective results to determine surface energy and we present its relationship with the spectroscopic properties. Herein, we propose a new experimental model to quantify the effects of concentration-dependent and surface properties.

**Figure 8:**
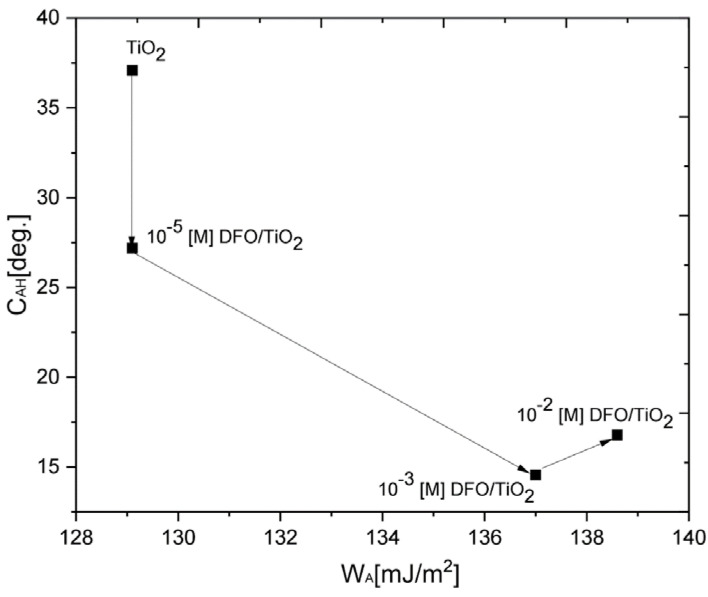
Contact angle hysteresis (C_AH_) as a function of work of adhesion W_A_ for a variety of concentrations of DFO in TiO_2_ thin films in contact with water.

The studied surfaces, both with CNWs and without, exhibited wettability characteristics of hydrophilic substrata (θ_ϒ_[deg.] < 90°) (Table 2). The surface modification effect was reflected in the following variability of the C_AH_ parameters: θ_A_[deg.] decreased almost twice the time for DFO/TiO_2_ samples and a few percent for DFO/TiO_2_/CNWs and θ_R_[deg.], only a few percent for both types of samples, and π [mN/m] decreased also with increasing concentration of DFO, but for CNW samples, have high values and more stable values with the increasing concentration of DFO than for only TiO_2_ thin films. ϒ_SV_ [mJ/m^2^] and W_A_ [mJ/m^2^] increased by a few percent with the increasing of concentration of DFO, and for TiO_2_/CNW samples, we observed a more stable value. As a result, the studied surface after the treatment procedure becomes more hydrophilic. The total surface free energy decrease results from a drop in the dispersive interaction component as it was found here. The observed ϒ_SV_ [mJ/m^2^] from 53.10 to 66.89 (DFO/TiO_2_) and 60.46 to 63.57 (DFO/TiO_2_/CNWs) may be also attributed to the adsorption at the solid surface. ϒ_SV_ [mJ/m^2^] is rather high and interactions between the substratum and water molecules are rather strong, γ_SV_^d^/γ_SV_ = 0.89–0.97. The value of C_AH_ was chosen as a parameter that significantly correlates with the changes in the matrices related to DFO dye concentration variability. Particularly, as the DFO concentration increases, the C_AH_ value decreases, indicating an increase in structure ordering when aggregates appear in the TiO_2_ matrix (Figure 8); unfortunately, such regular changes were not observed in the TiO_2_/CNW matrix. Architecture and aggregate orientation in reference to the surface can be derived from the cross-sectional limiting area A_lim_ occupied by the adsorbed unit, related to the 2D adsorptive film pressure Π: A_lim_ = RT/Π, according to Equation (2). The ratios of the limiting areas (referred to TiO_2_ case) were as follows, for a variety of concentrations: 1.72 (10^−5^ [M]), 3.85 (10^−3^ [M]), 3.89 (10^−2^ [M]). The more expanded spatial aggregation structures were noticed for less diluted solutions.

The complex surface wettability evolution of the model substrata can be presented in the spatial distribution of the experimental points placed in the 2D space of C_AH_ plotted versus W_A_ [1,2]. CAH reflects two surface features (roughness and spatial heterogeneity of adsorbed material), whereas W_A_ stands for the strength of the adhesion forces to the substratum. As is evident from data in Figure 8, starting from the reference clean surface case (TiO_2_), the surface for the most diluted system (10^−5^ [M]) become smoother and more homogenous (C_AH_ ↓ and W_A_~ constants). The concentration increase resulted in further increasing homogeneity (C_AH_ ↓ being of the order of 15.0–16.8 mN m^−1^), but the surface adhesion strength was significantly intensified (W_A_ evolved from 129 to 137–138.6 mJ m^−2^).

### 3.4. Quantum Chemistry Calculations

**Figure 9:**
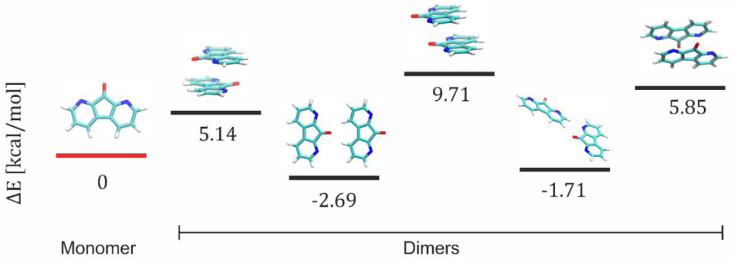
The energy differences between a system composed of monomers and one containing clustered molecules in the form of second aggregates (dimer).

To investigate the propensity of DFOs to form aggregates, we performed density functional theory (DFT) calculations on several systems of different levels of complexity. We estimated the energy differences between a system composed of monomers and one containing clustered molecules in the form of second- and third-order aggregates. The simplest form of aggregation is the formation of dimers. This is where our analysis began. By analyzing five different spatial configurations of DFOs forming a dimer (see Figure 9), we calculated the difference between the internal energy of the complex of molecules and the sum of the energies of the individual components in the monomer form. In two cases out of five, we obtained energies lower than the initial monomer energy, suggesting that DFO with a suitable arrangement of molecules is prone to form aggregates. DFT calculations suggest that the planar arrangement of molecules (second and fourth case) is more energetically favorable than sandwich-like configurations. However, one should remember that this is a simplified model of aggregation, which does not take into account the influence of the environment.

**Figure 10:**
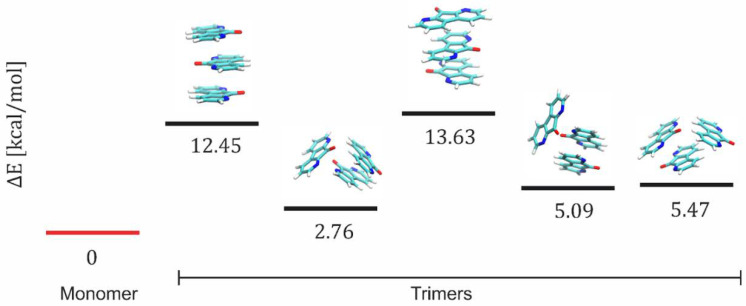
The energy differences between a system composed of monomers and one containing clustered molecules in the form of third-order aggregates (trimer).

In the next step, we analyzed higher-order aggregates—the trimers (see Figure 10). In the case of trimers, each of the five systems analyzed turned out to be less energetically favorable than the three separate monomers. However, of the five cases, aggregates with unconventional/unordered configurations (second, fourth, and fifth configurations in Figure 10) were more energetically favorable than sandwich-like ones (Figure 10, first and third configuration). Even though the calculations in a vacuum are a rather coarse approximation, they show the tendency of DFO itself to aggregate.

## 4. Conclusions

The acquired parameters characterizing the spectroscopic and surface properties led us to answer the previously asked question: Do the concentration modifications of the dye in a titanium dioxide matrix and a titanium dioxide–carbon nanowalls hybrid matrix correlate with the changes in the surface energy and the mode of interaction on the surface of these materials with the external environment?

The thin DFO/TiO_2_ film where the matrix is in the form of titanium dioxide affects the system’s ordering. As a result, the unconventional aggregates of DFO are formed, which changes the emission capability of the system. The presence and ordering of the system were further confirmed by contact angle hysteresis. The C_AH_ value significantly decreases with the increase in dye:DFO concentration, which indicates a uniform distribution of the dye on the surface of the thin film. The aggregation process leads to the uniform distribution of DFO dye on the surface. Thus, parameters such as contact hysteresis can be used to describe the increase in the aggregation process, but only for DFO/TiO_2_ thin films. Aggregation processes revealed by spectroscopic methods and analysis of physicochemical parameters resulting from wetting angle measurements are compatible only in the case of DFO/TiO_2_ system characterization. In the case of DFO/TiO_2_/CNWs, we observe no dependence of C_AH_ on dye concentration change. The observed dependence is not ordered and homogeneous. It is likely that the surface energy of CNWs significantly affects the aggregation processes of DFO in the TiO_2_/CNW system, even causing the extinction of the emission of the formed aggregates in these matrices.

In addition, using the quantum chemical calculations, we confirmed the energetic tendency of the DFO molecule to aggregate with a preference for the dimer structure formation. This result is strongly compatible with our recent publication results [21,22,23], where we confirmed the aggregation ability of DFO in ethanol and the emission of DFO aggregates in ethanol with an emission maximum at a similar wavelength as the DFO complex with glycine (an alpha-amino acid). These results significantly affect the application of DFO in Friction Ridge Analysis and are predictive of the application of DFO on different types of substrates with different surface energy characteristics.

As a final product, a biomaterial was obtained in which the aggregation processes of biologically active molecules can be controlled and used to reveal, among others, alpha-amino acids, which are important markers for many biochemical processes occurring in living organisms.

## Figures and Tables

**Figure 1:**
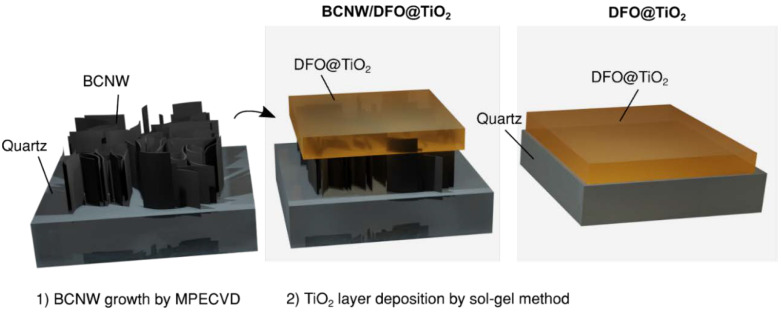
The scheme of obtaining DFO/TiO_2_ and DFO/TiO_2_/CNW thin films.

**Table 1:** Ellipsometrically estimated properties of DFO/TiO_2_ [21] films related to DFO/TiO_2_/CNW films with different concentrations of DFO dye.

c [M] DFO/TiO_2_ or TiO_2_/CNWs	n _(TiO2)_	d _(TiO2)_ [nm]	n _(DFO/TiO2/CNW)_	d _(DFO/TiO2/CNW)_ [nm]
0 [M]	1.514	300	2.32/1.76	135/533
10^−5^ [M]	1.592	386	- */1.77	30/520
10^−2^ [M]	1.624	356	2.58/1.75	90/530

* not measurable due to high roughness.

**Table 2:** Wettability parameters (mean values) of studied solid substrata in contact with distilled water, ϒ_LV_ = 72.77, T = 23.3 °C, Wc = 145.54 mJ/m^2^.

C_DFO_ [mol/dm^3^]	θ_ϒ_ [deg.]	θ_A_ [deg.]	θ_R_ [deg.]	C_AH_ [deg.]	Π [mN/m]	ϒ_SV_ [mJ/m^2^]	W_A_ [mJ/m^2^]	ϒ^SVd^ [mJ/m^2^]	ϒ^d^/ϒ_SV_ [a.u.]
**TiO_2_**	37.67	51.89	14.79	37.10	25.4	53.10	117.7	47.58	0.896
**10^−5^ DFO/TiO_2_**	13.04	39.25	12.06	27.19	14.86	61.06	129.1	57.28	0.938
**10^−3^ DFO/TiO_2_**	21.01	28.07	13.49	14.58	6.6	66.89	137.0	64.46	0.964
**10^−2^ DFO/TiO_2_**	10.49	25.16	8.36	16.8	6.1	67.82	138.6	66.03	0.974
**TiO_2_/CNWs**	24.45	40.5	14.13	26.37	15.2	60.46	128.1	56.38	0.933
**10^−5^ DFO/TiO_2_/CNWs**	29.31	46.76	12.92	33.84	21.1	56.46	122.6	51.66	0.915
**10^−3^ DFO/TiO_2_/CNWs**	15.43	34.71	11.99	22.72	11.4	63.57	132.6	60.40	0.950
**10^−2^ DFO/TiO_2_/CNWs**	21.72	38.95	10.20	28.75	15.0	61.13	129.4	57.49	0.940

## Data Availability

Not applicable.

## References

[1] Yusuf M.M., Imai H., Hirashima H. (2002). Preparation of porous titania film by modified sol-gel method and its application to photocatalyst. J. Sol-Gel Sci. Technol..

[2] Xagas A.P., Androulaki E., Hiskia A., Falaras P. (1999). Preparation, fractal surface morphology and photocatalytic properties of TiO_2_ films. Thin Solid Films.

[3] Yu J., Yu J.C., Cheng B., Zhao X., Zheng Z., Li A.S.K. (2002). Atomic force microscopic studies of porous TiO_2_ thin films prepared by the sol-gel method. J. Sol-Gel Sci. Technol..

[4] Fabes B.D., Birnie D.P., Zelinski B.J.J. (1995). Porosity and composition effects in sol-gel derived interference filters. Thin Solid Films.

[5] Carbonaro C.M. (2011). Tuning the formation of aggregates in silica–Rhodamine 6G hybrids by thermal treatment. J. Photochem. Photobiol. A Chem..

[6] Vogel R., Meredith P., Kartini I., Harvey M., Riches J.D., Bishop A., Heckenberg N., Trau M., Rubinsztein-Dunlop H. (2004). Absorption and fluorescence spectroscopy of rhodamine 6G in titanium dioxide nanocomposites. Spectrochim. Acta Part A Mol. Biomol. Spectrosc..

[7] Vogel R., Meredith P., Kartini I., Harvey M., Riches J.D., Bishop A., Heckenberg N., Trau M., Rubinsztein-Dunlop H. (2003). Mesostructured Dye-Doped Titanium Dioxide for Micro-Optoelectronic Applications. ChemPhysChem.

[8] Jelley E.E. (1936). Spectral absorption and fluorescence of dyes in the molecular state. Nature.

[9] Kasha M., Rawls H.R., El-Bayoumi M.A. (1965). The exciton model in molecular spectroscopy. Pure Appl. Chem..

[10] Bojarski P., Matczuk A., Bojarski C., Kawski A., Kukliński B., Zurkowska G., Diehl H. (1996). Fluorescent dimers of rhodamine 6G in concentrated ethylene glycol solution. Chem. Phys..

[11] Gruszecki W.I. (1991). Structural characterization of the aggregated forms of violaxanthin. J. Biol. Phys..

[12] Inglot K., Martyński T., Bauman D. (2009). Molecular organization and aggregation in Langmuir and Langmuir-Blodgett films of azo dye/liquid crystal mixtures. Opto-Electron. Rev..

[13] McRae E.G., Kasha M. (1958). Enhancement of phosphorescence ability upon aggregation of dye molecules. J. Chem. Phys..

[14] Rohatgi K.K. (1968). Absorption spectra of the dimers of ionic dyes. J. Mol. Spectrosc..

[15] Monahan A.R., Blossey D.F. (1970). Aggregation of arylazonaphthols. I. Dimerization of Bonadur Red in aqueous and methanolic systems. J. Phys. Chem..

[16] Somsen O.J., van Grondelle R., van Amerongen H. (1996). Spectral broadening of interacting pigments: Polarized absorption by photosynthetic proteins. Biophys. J..

[17] Kunzler J., Samha L., Zhang R., Samha H. (2011). Investigation of the effect of concentration on the molecular aggregation of cyanine dyes in aqueous solution. Am. J. Undergrad. Res..

[18] Antonov L., Gergov G., Petrov V., Kubista M., Nygren J. (1999). UV–Vis spectroscopic and chemometric study on the aggregation of ionic dyes in water. Talanta.

[19] Bojarski P. (1997). Concentration quenching and depolarization of rhodamine 6G in the presence of fluorescent dimers in polyvinyl alcohol films. Chem. Phys. Lett..

[20] Tyurin O.V., Bercov Y.M., Zhukov S.O., Levitskaya T.F., Gevelyuk S.A., Doycho I.K., Rysiakiewicz-Pasek E. (2010). Aggregation of dyes in the porous glass. Opt. Appl..

[21] Lewkowicz A., Bogdanowicz R., Bojarski P., Pierpaoli M., Gryczyński I., Synak A., Mońka M., Karczewski J., Struck-Lewicka W., Wawrzyniak R. (2020). The luminescence of 1,8-diazafluoren-9-one/titanium dioxide composite thin films for optical application. Materials.

[22] Zygadło P., Lewkowicz A. (2021). Structural-spectroscopic analysis of DFO/PVA films as potential materials used in revealing fingerprints on non-porous surfaces. Issue Forensic Sci..

[23] Lewkowicz A., Kantor M., Zalewski W., Bojarski P., Mońka M. (2022). Spectroscopic evidence of fluorescence by 1,8-diazafluoren-9-one aggregates—A prospective new ultrasensitive method for fingerprint trace detection. J. Forensic Sci..

[24] Strobel M., Lyons C.S. (2011). An essay on contact angle measurements. Plasma Processes Polym..

[25] Chibowski E. (2003). Surface free energy of a solid from contact angle hysteresis. Adv. Colloid Interface Sci..

[26] Niedziałkowski P., Ossowski T., Zięba P., Cirocka A., Rochowski P., Pogorzelski S.J., Ryl J., Sobaszek M., Bogdanowicz R. (2015). Poly-l-lysine-modified boron-doped diamond electrodes for the amperometric detection of nucleic acid bases. J. Electroanal. Chem..

[27] Pierpaoli M., Ficek M., Jakóbczyk P., Karczewski J., Bogdanowicz R. (2021). Self-assembly of vertically orientated graphene nanostructures: Multivariate characterization by Minkowski functionals and fractal geometry. Acta Mater..

[28] Pierpaoli M., Ficek M., Rycewicz M., Sawczak M., Karczewski J., Ruello M.L., Bogdanowicz R. (2019). Tailoring electro/optical properties of transparent boron-doped carbon nanowalls grown on quartz. Materials.

[29] Kubicki A.A., Bojarski P., Grinberg M., Sadownik M., Kukliński B. (2006). Time-resolved streak camera system with solid-state laser and optical parametric generator in different spectroscopic applications. Opt. Commun..

[30] Pogorzelski S.J., Berezowski Z., Rochowski P., Szurkowski S. (2012). A novel methodology based on contact angle hysteresis approach for surface changes monitoring in model PMMA-Corega Tabs system. Appl. Surf. Sci..

[31] Extrand C.W., Kumagai Y. (1995). Liquid Drops on an Inclined Plane: The Relation between Contact Angles, Drop Shape, and Retentive Force. J. Colloid Interface Sci..

[32] Mazurek A.Z., Pogorzelski S.J., Boniewicz-Szmyt K. (2009). Adsorption of natural surfactants present in sea waters at surfaces of minerals: Contact angle measurements. Oceanologia.

[33] Pogorzelski S., Boniewicz-Szmyt K., Grzegorczyk M., Rochowski P. (2022). Wettability of Metal Surfaces Affected by Paint Layer Covering. Materials.

[34] Weigend F., Ahlrichs R. (2005). Balanced basis sets of split valence, triple zeta valence and quadruple zeta valence quality for H to Rn: Design and assessment of accuracy. Phys. Chem. Chem. Phys..

[35] Grimme S., Antony J., Ehrlich S., Krieg H. (2020). A consistent and accurate ab initio parametrization of density functional dispersion correction (DFT-D) for the 94 elements H-Pu. J. Chem. Phys..

[36] Pierpaoli M., Lewkowicz A., Rycewicz M., Szczodrowski K., Ruello M.L., Bogdanowicz R. (2020). Enhanced photocatalytic activity of transparent carbon nanowall/TiO2 heterostructures. Mater. Lett..

[37] Hong Y., Lam J.W., Tang B.Z. (2011). Aggregation-induced emission. Chem. Soc. Rev..

[38] Zhao Z., Zhang H., Lam J.W., Tang B.Z. (2020). Aggregation-induced emission: New vistas at the aggregate level. Angew. Chem. Int. Ed..

[39] Mei J., Hong Y., Lam J.W., Qin A., Tang Y., Tang B.Z. (2014). Aggregation-induced emission: The whole is more brilliant than the parts. Adv. Mater..

[40] Kang M., Zhang Z., Song N., Li M., Sun P., Chen X., Wang D., Tang B.Z. (2020). Aggregation-enhanced theranostics: AIE sparkles in the biomedical field. Aggregate.

[41] Sebastian E., Philip A.M., Benny A., Hariharan M. (2018). Inside Cover: Null Exciton Splitting in Chromophoric Greek Cross (+) Aggregate (Angew. Chem. Int. Ed. 48/2018). Angew. Chem. Int. Ed..

[42] Luo J., Xie Z., Lam J.W., Cheng L., Chen H., Qiu C., Kwok H.S., Zhan X., Liu Y., Zhu D. (2001). Aggregation-induced emission of 1-methyl-1, 2, 3, 4, 5-pentaphenylsilole. Chem. Commun..

[43] Yang J., Fang M., Li Z. (2020). Organic luminescent materials: The concentration on aggregates from aggregation-induced emission. Aggregate.

[44] Gindl M., Sinn G., Gindl W., Reiterer A., Tschegg S. (2001). A comparison of different methods to calculate the surface free energy of wood using contact angle measurements. Colloids Surf. A Physicochem. Eng. Asp..

[45] Gast A.P., Adamson A.W. (1997). Physical Chemistry of Surfaces.

[46] Rodrıguez-Valverde M.A., Cabrerizo-Vılchez M.A., Rosales-Lopez P., Paez-Duenas A., Hidalgo-Alvarez R. (2002). Contact angle measurements on two (wood and stone) non-ideal surfaces. Colloids Surf. A Physicochem. Eng. Asp..

[47] Chibowski E. (2007). On some relations between advancing, receding and Young’0027s contact angles. Adv. Colloid Interface Sci..

